# Effects of the COVID-19 Pandemic on Breastfeeding Initiation and Duration: A Retrospective Cohort Study

**DOI:** 10.7759/cureus.54231

**Published:** 2024-02-15

**Authors:** Mariana M Meneses, Catarina Freitas, Joana Machado Morais, Maria S Dias, Cláudia Ferraz, Sara Peixoto

**Affiliations:** 1 Serviço de Pediatria, Hospital Pedro Hispano, Porto, PRT

**Keywords:** upper respiratory infections, neonatal intensive care unit (nicu), newborn health, breastfeeding duration, covid-19 pandemic

## Abstract

Introduction and objectives: Several studies support the health benefits of breastfeeding for both the mother and the newborn. However, a significant number of mothers discontinue breastfeeding within the first six months of childbirth, with several factors influencing breastfeeding adherence. The purpose of this study is to assess the impact of the COVID-19 pandemic on the prevention of mother-to-newborn infection transmission, breastfeeding patterns and duration, and the incidence of other infections during the first year of life.

Methods: Data from a sample of 39 mothers who gave birth at the Hospital Pedro Hispano in Porto, Portugual, between March 2020 and November 2021 were collected and a telephone questionnaire was administered. Statistical analysis was conducted using R software, v. 4.2.1 (R Foundation for Statistical Computing, Vienna, Austria).

Results and discussion: In terms of the impact of the COVID-19 norm 18/2020, which went into effect on March 30th, our research found that the type of feeding during hospitalization was significantly influenced by this norm (X^2^=10.30, p=0.006). We also confirmed that mothers who received home assistance breastfed for an extra 4.5 months (95% CI: 1-7.5) compared with mothers who did not receive such assistance. Regarding the effect of COVID-19 and breastfeeding on newborn health, our study found that if the total duration of breastfeeding is less than six months, an infection is approximately five times more likely (95% CI = 1.06- 29.56).

Conclusion: Overall, the findings of this study indicate that the efforts implemented at Hospital Pedro Hispano to limit the effects of the COVID-19 pandemic had some effect on immediate breastfeeding patterns, but not on the total duration of breastfeeding or newborn health. Nonetheless, more continuous assistance at home would have been beneficial.

## Introduction

According to the nutrition commissions (ESPGHAN, 2017; SPP nutrition commission, 2012), the World Health Organization (WHO, 2009), and the American Academy of Paediatrics, exclusive breastfeeding during the first six months of life is the primary method of infant feeding, which should be incentivized and continued after medical release. Several studies support the benefit to the health of both the newborn and mother [1-5]. One of the most frequently mentioned benefits is a reduction in the occurrence of infections [6]. Yet, a large majority of mothers discontinue breastfeeding at the first six months of life, with numerous factors influencing adherence to breastfeeding [6].

Infection with the SARS-CoV-2 virus became a pandemic in March 2020, and very little was known about the harmful consequences on neonates at the time. Because of the uncertainty around the possibility of transmission, preventative measures were implemented, with mother-child separation after childbirth being a contentious topic as the risk of infection of the baby was evaluated against the benefits of early bonding and breastfeeding. As a result, it was up to the health institutions to make their own decisions based on the mother's wishes, the hospital's facilities, and the availability of health personnel.

Because breastfeeding is dependent on the mother-child bond, which is dependent on skin-to-skin contact at birth and rooming-in after childbirth, the goal of this study is to assess the impact of the COVID-19 pandemic, specifically, the measures implemented at the Hospital Pedro Hispano (Porto, Portugal), regarding the prevention of infection transmission from mother to newborn, breastfeeding patterns and duration, and the incidence of other infections.

## Materials and methods

A sample of 39 mothers who gave birth at Hospital Pedro Hispano between March 2020 and November 2021 and tested positive for COVID-19 at least once in the month prior to childbirth completed a telephone survey; the mothers' verbal consent was obtained before four medical professionals recorded their responses. The questionnaire (Appendix 1) was comprehensive and contained a thorough definition and interpretation of various behavioural patterns and effects throughout three distinct times: gestation, immediately following childbirth and hospitalization, and the first few months of parental leave.

The registered replies were carefully examined and converted to appropriate variables: (a) dichotomous variables (such as the mother's intentions to breastfeed prior to childbirth, the COVID test result of the mother at birthdate, implementation of the COVID norm 18/22, skin-to-skin contact at birth, rooming-in after childbirth, and assistance after hospital discharge); (b) categorical variables (for instance, the type of feeding and mode of administration); and (c) quantitative variables (e.g., the total duration of breastfeeding in months). For assessing most hypotheses, it was considered useful to divide the data into five distinct (though not necessarily independent) breastfeeding moments, namely: postpartum, in the initial hours after birth, in internment, upon medical release, and during the first weeks at home.

In the first approach, associations between the various dichotomous and categorical variables throughout the distinct breastfeeding moments were tested with the chi-square test (Yates correction applied when appropriate). Afterwards, the influence of the type of feeding employed during each breastfeeding moment over the total duration of breastfeeding as well as the influence of the type of feeding in internment compared to the type of feeding during the first weeks at home were analysed with Wilcoxon rank sum and Kruskall-Wallis tests, respectively. Other factors such as the mother's intentions prior to childbirth, whether COVID norm 18/2022 had taken effect by the birthdate, the mode of administration of maternal milk (i.e., directly in the breast or through the baby bottle), and if the mother received home assistance or not, were also examined for their impact on the total duration of breastfeeding using Wilcoxon test. During this period, home assistance was influenced not only by the availability of professionals and limitations set out in the COVID norm 18/2022, but also by the mother's willingness to receive such care.

Finally, we explored all possible associations between several previously listed grouping and response variables related to the general health of the newborn (i.e. occurrence of some specific infections in the first year of life), by applying the chi-square test (with Yates correction when appropriate).

In all conducted statistical tests, a p-value threshold of 0.05 was used to reject the null hypothesis. R software, v. 4.2.1 (R Foundation for Statistical Computing, Vienna, Austria) was used for statistical analysis. The health ethics committee of ULS Matosinhos unanimously approved the study's implementation (no. 55/CES/JAS).

## Results

Exploratory analysis

This study focused on births that occurred at the Hospital Pedro Hispano between March 2020 and November 2021 and in which the mother tested positive for COVID-19 at least once during the month preceding the childbirth. As a result, a working sample size of 39 birth cases was established. Table 1 contains information on the general characterisation of the sample based on socio-demographic criteria such as the mother's age, literacy level, household size, parity, birth weight, and biological gender of the newborn.

**Table 1:** Summary demographics of the population of study. NB=newborn

		Frequency	Proportion
Civil State	Married	17	44%
	Single	13	33%
	Civil Union	9	23%
Level of Education	Basic education	4	10%
	High School	17	44%
	Graduate	11	28%
	Master or PhD	7	18%
Number of household members	1	3	8%
	2	11	28%
	3	15	38%
	4	8	21%
	6	2	5%
Parity	Multiparous	19	50%
	Primiparous	19	50%
Newborn gender	Female	23	59%
	Male	16	41%
Age of the mother	20-24	2	5%
	25-29	8	21%
	30-34	9	23%
	35-39	15	38%
	40-44	5	13%
Weight of NB at birth	< 2 Kg	3	8%
	2-2.5 Kg	4	9%
	2.5-3 Kg	12	31%
	3-3.5 Kg	17	44%
	3.5-4 Kg	3	8%

About 82% of mothers who tested positive for SARS-CoV-2 infection in the month preceding childbirth were still positive on the date of their babies' birth; among this group, 75% were asymptomatic, 22% had mild symptoms, and only 3% (one individual) had serious symptoms requiring hospitalization. COVID-19 was documented to have altered the kind of birthing option - eutocic or dystocic - in exactly six cases.

Influence of COVID-19 on breastfeeding patterns and consistency of feeding across different moments

To analyze the impact of pandemics on overall breastfeeding practices, five distinct moments were defined: breastfeeding postpartum; breastfeeding in the initial hours after birth; type of feeding during internment; type of feeding upon medical release; and type of feeding during the first weeks at home.

To test if the five stated moments are independent, we analyzed Kendall's Tau correlation for ordinal variables. Herein, we used the term "Mix" to denote caregivers who breastfed and supplemented with milk formula and to distinguish from those who provided exclusive breast milk (EBM) and exclusive milk formula (EMF); for instance, EMF=1, Mix=2, and EBM=3. The strongest correlation (0.747) was found between the type of feeding during internment and the type of feeding at medical release moments, followed by weaker correlations between the type of feeding at medical release and the type of feeding during the first weeks at home (0.676) and breastfeeding postpartum and type of feeding at medical release (0.676).

Concerning the COVID pandemic, whether the mother tested COVID-positive or negative on the birthdate had no effect on her decision to breastfeed postpartum (i.e., the COVID test result on the birthdate was not significantly associated with breastfeeding in the postpartum, according to chi-square test with Yates correction: X2=1.24x10-4, p=0.991). However, COVID was used to justify nine of the 14 cases in which the mother did not breastfeed afterwards (and five with other reasons).

Beginning March 30, Sars-CoV-2-positive mothers were urged to avoid skin-to-skin contact, breastfeeding, and sharing a room with their infant under COVID-19 regulation 18/2020. This proposal was followed by Pedro Hispano Hospital until the end of May 2020. Indeed, among the 39 mothers analyzed, only one out of every 13 newborns between March 30th and May 30th 2020 made skin-to-skin contact and were rooming-in post-partum.

**Figure 1:**
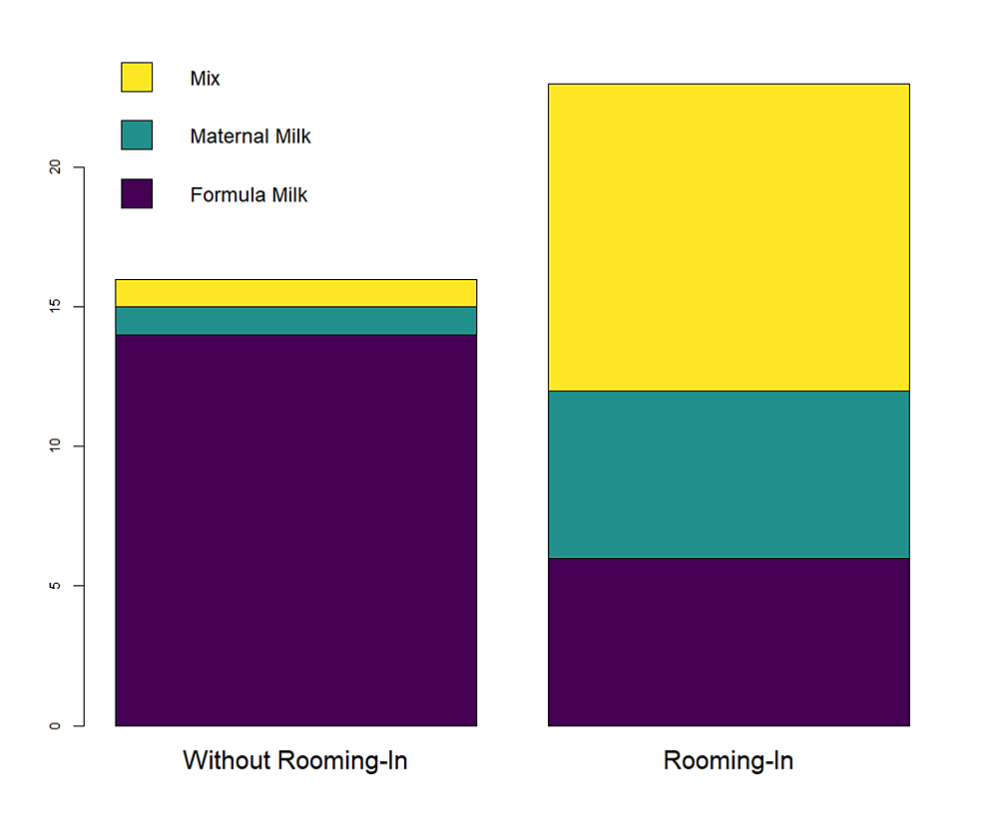
Type of feeding during hospitalization according to having done rooming-in or not.

We looked for significant connections between both the skin-to-skin practice after birth and the rooming-in of mothers with their newborn, and the times of "breastfeeding in the first hours after childbirth" and "kind of feeding during hospitalization". Skin-to-skin contact is significantly associated with EBM within the first hours of life (X2=12.36, p=0.001), and rooming-in is significantly connected with the type of feeding during hospitalization (X2= 14.31, p=0.001), as shown in Figure 1.

**Figure 2:**
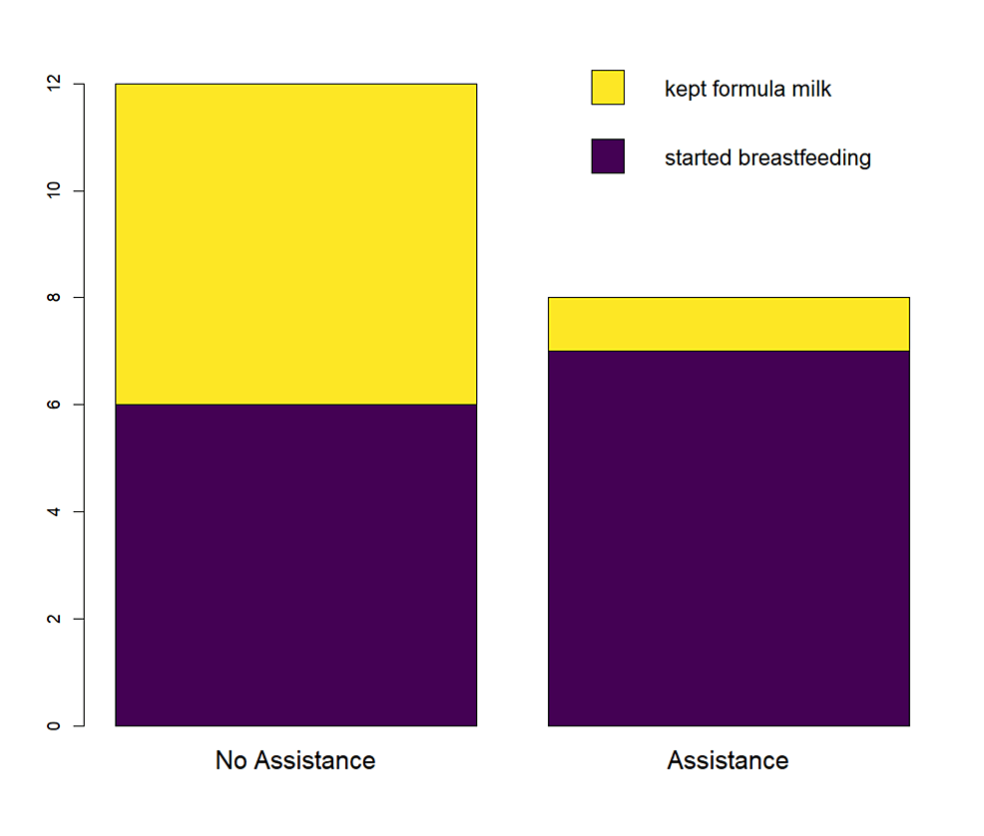
Barplot indicating whether mothers who were given exclusive formula milk during hospitalization (n=20) continued to give exclusive formula milk after leaving the hospital or breastfeeding (i.e., did EBM or Mix) at home, depending on whether they received support or not. Absolute frequencies are represented by bar heights.

We also addressed the impact of assistance after hospital discharge on the decision to type of feeding at medical release (either Mix or EBM) at home among mothers who during hospitalization did not breastfeed at all. We found that, with no assistance, only 50% of the mothers who did not introduce maternal milk / exclusively fed formula milk (n=20) during hospitalization started breastfeeding after hospital leave, compared to 87.5% of mothers who introduced maternal milk after hospital leave when receiving assistance (Figure 2). However, this difference is not statistically significant (X²=1.55, p=0.213, Yates correction applied).

Influence of the type of feeding during hospitalization and type of feeding during the first weeks at home over the total duration of breastfeeding

**Figure 3:**
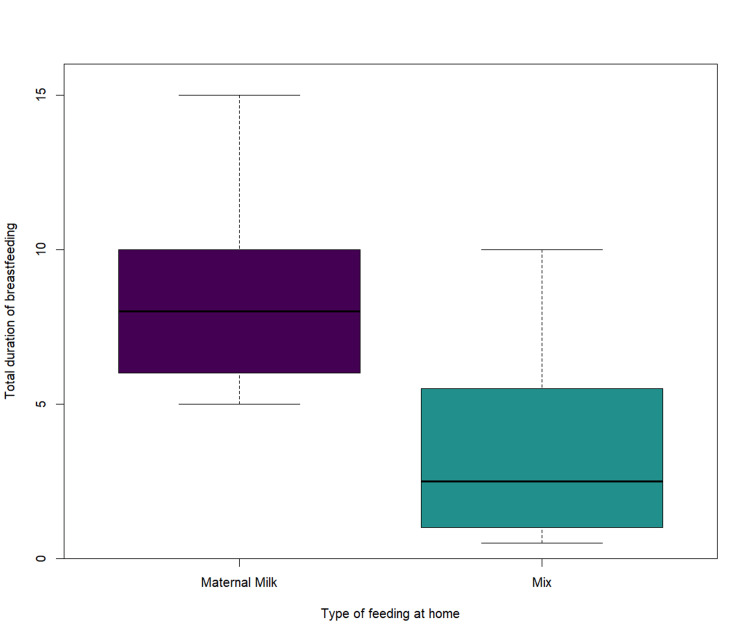
Box-plot representation of total duration of breastfeeding (in months) depending on the type of feeding during the first weeks at home.

Bearing in mind the nuanced independence of the five feeding moments (as demonstrated by the previously reported relatively low correlation scores among the moments), we then investigated the association of those with the entire duration (in months) of breastfeeding. The style of feeding during the first weeks at home directly influenced the total duration of breastfeeding (Wilcoxon rank sum test, W=207.5, p=0.0006) for a significance threshold of α=0.05, excluding cases where breastfeeding was practically non-existent (i.e. EFM at home; total sample size n=31). This is depicted in Figure 3, where we can see that when the feeding at home was purely EBM, the overall duration of breastfeeding was, on average, five months longer than when it was mixed.

Still regarding the same 31 mothers who breastfed at home, and separately analysing mothers who practised EBM vs. Mix, it was found that the type of feeding during hospitalization did not influence the total duration of breastfeeding (Kruskall-Wallis and Wilcoxon rank tests, p = 0.2152 and p = 0.6702 for EBM and Mix, respectively).

**Figure 4:**
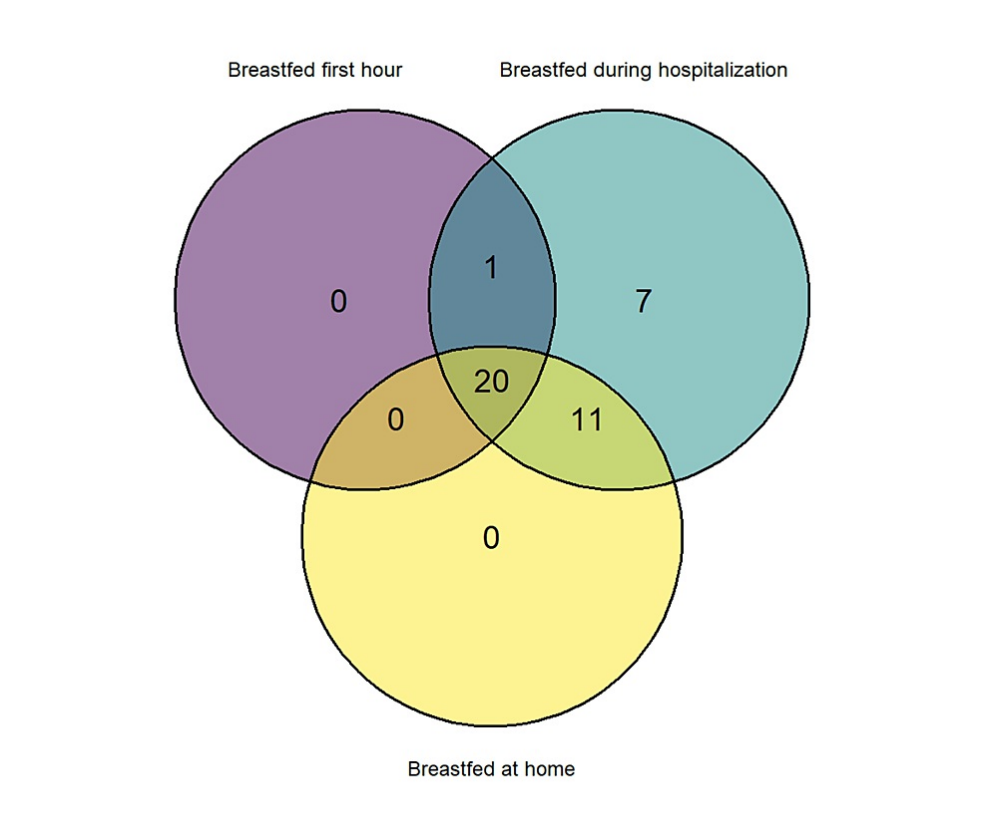
Venn diagram representation showing the overlap of mothers that breastfed (either EBM or Mix) in the first hour after birth (purple ellipse) during hospitalization (blue ellipse) and at home (yellow ellipse).

In terms of the effects of COVID-19 norm 18/2020, our research found that the kind of feeding during hospitalization was strongly influenced by this norm (X2=10.30, p=0.006). For example, mothers who gave birth between March and May 2020 utilized EFM at hospitalization 9.73 times more than mothers who did not (Fisher's exact test p=0.0057). Yet, as shown in the Venn diagram (Figure 4), mothers who had not nursed (either EBM or Mix) during the first hours of life and during hospitalization breastfed at home. However, the type of feeding during the first weeks at home does not appear to have been influenced by this standard (X2=0.49, p=0.813), indicating that following hospital discharge, mothers' choice of feeding (EBM, EFM, or Mix) was not directly affected by COVID-19 norm 18/2020.

Other factors that were examined for their impact on the total duration of breastfeeding included the mother's breastfeeding intentions prior to childbirth, COVID norm 18/2022 being effective or not on the birthdate, the mode of administration of maternal milk (i.e., directly in the breast or through the baby bottle) for mothers who either breastfed exclusively or mixed with EFM during hospitalization, and the mother receiving home assistance or not. Only the latter proved statistically significant (W = 72.5, p = 0.003), with mothers who received support during the first weeks at home nursing for 4.5 months longer (95% CI: 1-7.5) than mothers who did not.

Influence of COVID-19 and breastfeeding on the health of the new-born

We tested (with a chi-square test at a significance level of α=0.05, Yates correction when at least one expected value in contingency table <5) all possible associations between the dichotomous grouping and response variables described below to assess the impact of some COVID-related events and breastfeeding patterns and duration on the general health of the newborn:

Grouping Variables

Grouping variables included the following: (a) COVID test result on childbirth date (positive/negative); (b) type of feeding in the postpartum period (with/without maternal milk); (c) type of feeding in the first hours after childbirth (with/without maternal milk); (d) type of feeding during hospitalization (with/without maternal milk); (e) type of feeding at medical release (with/without maternal milk); (f) type of feeding during the first weeks at home (with/without maternal milk); (g) skin-to-skin practice after birth (yes/no); (h) health incidents during gestation (yes/no); (i) total duration of breastfeeding (classes <6 months / >6 months).

Response Variables

Response variables included the following: (a) infections post-partum in the first year of life (yes/no); (b) allergic/ atopic pathology (yes/no); (c) covid occurrence (yes/no; (d) otitis occurrence (yes/no); (d) laryngitis occurrence (yes/no); (e) bronchiolitis occurrence (yes/no); (f) respiratory tract infection occurrence (yes/no); (g) urinary tract infections occurrence (yes/no).

There were just two significant connections found between these categories and response factors. The first was a relationship between the total time of breastfeeding and the overall occurrence of infections post-partum (X2= 5.61, p= 0.018). Indeed, the relative risk of infections in the newborn is 1.82 times greater when the total duration of breastfeeding (either Mix or EBM) is less than six months, with an odds ratio of five (95% CI = 1.06 - 29.56) indicating that infections in the first year of life are approximately five times more likely when the total duration of breastfeeding (either Mix or EBM) is less than six months. Also, a connection was discovered between the type of feeding during the hospitalization and the incidence of respiratory tract infection (X2 = 3.91.048).

## Discussion

This study set out five moments to assess the impact of the COVID-19 pandemic on breastfeeding patterns and the potential effects on children's health in the first year of life. We must remember that the connections for these moments are not regarded to be sufficiently definitive, and so we can conclude that there is a degree of independence regarding the feeding type throughout the five instances. It is worth noting, however, that all the mothers in our dataset who exclusively nursed while hospitalized continued to exclusively breastfeed at home. This shows that in a bigger sample, there could have been a higher association between the kind of feeding during hospitalization and at home and that mothers are unlikely to discontinue nursing at home if the breastfeeding regimen was already established during hospitalization [6,7].

Regarding the effects of COVID-19 norm 18/2020, our research found that the kind of feeding during hospitalization was strongly influenced by this norm [8]. However, the type of feeding during the first weeks at home does not appear to have been influenced by this standard, indicating that following hospital discharge, mothers' choice of feeding (EBM, EFM, or Mix) was not directly affected.

Another interesting point of debate is, that among mothers who did not breastfeed at all during their hospitalization, being supported after leaving the hospital may have influenced the decision to use a specific type of breastfeeding for medical release (either Mix or EBM) at home. However, a larger sample size would be required to confirm this hypothesis, especially since it appears that assistance increased after May 2020, i.e., during the most critical period when COVID-19 norm 18/2020 was being implemented, there was approximately five times less assistance after hospital discharge than afterwards (Fisher's exact test, p=0.033)

Concerning the influence of feeding type during hospitalization and at home on total breastfeeding duration, this study found that the type of feeding during hospitalization has no direct effect on total breastfeeding duration, despite the previously observed fact that mothers who exclusively breastfed during hospitalization had a much greater tendency to keep EBM at home and thus achieve longer total breastfeeding durations [6,8]. Furthermore, mothers who received home assistance breastfed for 4.5 months longer (95% CI: 1-7.5) than mothers who did not get assistance. One might wonder to what extent assiduous help would have successfully and significantly introduced nursing habits at home for mothers afflicted by the COVID-19 regulation limits.

Focusing on the impact of COVID-19 and nursing on newborn health, our study found that if the total period of breastfeeding (either Mix or EBM) is less than six months, infections are more likely (X2=5.61, p=0.018) to develop. Contrary to expectations, when no breast milk was administered during hospitalization, no respiratory tract infection occurred. However, not only are these findings based on a small sample size (four cases of respiratory tract infection versus 35 cases without illness), but also the limits enforced during the pandemic have resulted in a significant decrease in the incidence of respiratory illnesses in infants [9]. Further information is necessary.

## Conclusions

Notwithstanding the study's limitations (small sample size, retrospective nature of the study), the COVID-19 norm 18/2020 had a substantial influence on the type of feeding during hospitalization. However, among mothers who did not breastfeed at all during hospitalization, support after discharge was a decisive factor in the type of feeding they continued to do at home, especially among those who did not exclusively breastfeed in the hospital, thus enhancing breastfeeding at home and minimizing the impact of restrictions.

Contrary to predictions, there were no cases of respiratory tract infection that developed when breast milk was not given while the newborn was in the hospital. However, more studies are required.

## Appendices

Appendix 1

The table below comprises the questionnaire given to the study cohort (Table 1).

**Table 2:** Questionnaire

Appendix 1:
Age of the mother:
Civil status:
Single
Married
Consensual Union
Divorced
Widow
Academic qualifications
Elementary School
High School
Graduation
Master's/PhD
Family household:
Parity
Primiparous
Multiparous
Date of gestation onset:
Pregnancy Watched?
Yes
No
Preparation classes for childbirth?
Yes
No
In prenatal care was breastfeeding addressed?
Yes
No
Were you aware of the advantages for the newborn of breastfeeding?
Yes
No
Date of positive test:
Test positive at the time of delivery?
Yes
No
Desired pregnancy?
Yes
No
Any complications during pregnancy?
Yes
No
Duration of pregnancy:
< 35 weeks
35-37 weeks
37-40 weeks
> 40 weeks
Type of delivery:
Eutocic
Dystocia by sucker
Forceps dystocia
Cesarean section
Suction cup + cesarean section
Cesarean section + cup
Suction cup + forceps
Within the context of the pandemic COVID feels that the care provided during delivery at HPH was sufficient to minimize risk of transmission:
Yes
No
Do you feel that this care was necessary?
Yes
No
Gender of newborn
Female
Male
Apgar score:
Weight at birth:
Perinatal complications?
Yes
No
Did you intend to breastfeed before delivery?
Yes
No
Previous breastfeeding experience?
Yes
No
If yes, did you breastfeed exclusively until 6 months?
Yes
No
If no, at what age did you stop doing exclusive LM:
Did you consider your previous experience positive?
Yes
No
During this pregnancy, did you feel motivated to breastfeed after delivery?
Yes
No
If you did not breastfeed, please indicate the reason:
Suspected galctosemia
Active tuberculosis
HIV positive
HTLV-1 or -2 positive
Under anti-neoplastics, immunosuppressants, lithium, chloramphenicol, radiopharmaceuticals, iodine, bromocriptine
Maternal option
Previous negative experience
COVID19-related reasons
Not applicable
After delivery, did you skin to fur in the first hour of life?
Yes
No
Breast milk in the first hour of life?
Yes
No
If dairy formula in the 1st hour of life, was it due to COVID19?
Yes
No
Not applicable
After delivery, did you stay in rooming-in with the newborn?
Yes
No
If no, why?
COVID19
Maternal complications
Neonatal complications
Use of formula milk during hospitalization:
Yes
No
If yes, what is the reason?
Maternal hypogalacteal
Ponderal loss > 10% birth weight
Jaundice
Difficulties breastfeeding
Maternal complications
Other complications NN
If LM, was it the feed or bottle?
Maternal breast
Bottle
Maternal breast + bottle
If bottle, what is the reason?
Minimization of contact due to COVID19 by indication of health professional
Minimization of contact due to COVID19 by meternal option
Difficulties in technique
Local complications such as pain, cracks, or mastitis
During hospitalization, did you feel supported by your healthcare provider for breastfeeding?
Yes
No
Type of breastfeeding at the time of discharge?
Exclusive breast milk
Mixed breastfeeding
Exclusive milk formula
What type of feeding was given at home in the first weeks?
Exclusive breast milk
Mixed breastfeeding
Exclusive formula
Did the pandemic have any influence on this decision?
Yes
No
Were you housed together after discharge?
Yes
No
If you decided to breastfeed and were discharged with exclusive LM, until what age did you do exclusive breastfeeding?
What were the biggest difficulties you found when breastfeeding?
Difficulties in technique
Local complications
Doubts regarding the quantity and quality of the milk
Anatomical variations
Reason for giving up breastfeeding:
Maternal notion of hypogalactea
Doubts about the quality of the LM
Return to work
Local complications such as pain, fissures, or mastitis
Difficulties in maintaining isolation because of COVID19
Difficulties maintaining hygiene measures because of COVID19
Not applicable
After discharge from the maternity ward, did you feel accompanied and supported by the Health Professionals in your breastfeeding doubts?
Yes
No
Did COVID19 have an impact on how you felt regarding the previous question?
Yes
No
Child's current age (in months):
Since birth, have the baby had any infectious events?
Yes
No
If yes, was it acute otitis media?
Yes
No
If yes, was it infection of the lower VA by RSV?
Yes
No
If yes, was it infection of the lower VA by Strepto pneumoniae?
Yes
No
If yes, was it Haemophilus influenza infection?
Yes
No
If other, please indicate here:
Does the baby has allergic/atopic pathology?
Yes
No
Did the baby need hospitalization?
Yes
No
